# Enhancement of median nerve regeneration by mesenchymal stem cells engraftment in an absorbable conduit: improvement of peripheral nerve morphology with enlargement of somatosensory cortical representation

**DOI:** 10.3389/fnana.2014.00111

**Published:** 2014-10-16

**Authors:** Julia T. Oliveira, Ruben Ernesto Bittencourt-Navarrete, Fernanda M. de Almeida, Chiara Tonda-Turo, Ana Maria B. Martinez, João G. Franca

**Affiliations:** ^1^Laboratório de Neurodegeneração e Reparo, Instituto de Ciências Biomédicas, Centro de Ciências da Saúde, Universidade Federal do Rio de JaneiroRio de Janeiro, Brazil; ^2^Departamento de Fisiologia, Instituto de Ciências Biológicas, Universidade Federal de Juiz de ForaJuiz de Fora, Brazil; ^3^Universidade Federal do Rio de JaneiroMacaé, Brazil; ^4^Department of Mechanical and Aerospace Engineering, Politecnico di TorinoTorino, Italy; ^5^Departamento de Anatomia Patológica, Faculdade de Medicina, e Pós Graduação em Anatomia Patológica, Centro de Ciências da Saúde, Hospital Universitário Clementino Fraga Filho, Universidade Federal do Rio de JaneiroRio de Janeiro, Brazil; ^6^Programa de Neurobiologia, Instituto de Biofísica Carlos Chagas Filho, Centro de Ciências da Saúde, Universidade Federal do Rio de JaneiroRio de Janeiro, Brazil

**Keywords:** cortical plasticity, median nerve lesion, stem-cell therapy, polycaprolactone conduits, nerve regeneration, rat

## Abstract

We studied the morphology and the cortical representation of the median nerve (MN), 10 weeks after a transection immediately followed by treatment with tubulization using a polycaprolactone (PCL) conduit with or without bone marrow-derived mesenchymal stem cell (MSC) transplant. In order to characterize the cutaneous representation of MN inputs in primary somatosensory cortex (S1), electrophysiological cortical mapping of the somatosensory representation of the forepaw and adjacent body parts was performed after acute lesion of all brachial plexus nerves, except for the MN. This was performed in ten adult male Wistar rats randomly assigned in three groups: *MN Intact* (*n* = 4), *PCL-Only* (*n* = 3), and *PCL+MSC* (*n* = 3). Ten weeks before mapping procedures in animals from *PCL-Only* and *PCL+MSC* groups, animal were subjected to MN transection with removal of a 4-mm-long segment, immediately followed by suturing a PCL conduit to the nerve stumps with (*PCL+MSC* group) or without (*PCL-Only* group) injection of MSC into the conduit. After mapping the representation of the MN in S1, animals had a segment of the regenerated nerve processed for light and transmission electron microscopy. For histomorphometric analysis of the nerve segment, sample size was increased to five animals per experimental group. The *PCL+MSC* group presented a higher number of myelinated fibers and a larger cortical representation of MN inputs in S1 (3,383 ± 390 fibers; 2.3 mm^2^, respectively) than the *PCL-Only* group (2,226 ± 575 fibers; 1.6 mm^2^). In conclusion, MSC-based therapy associated with PCL conduits can improve MN regeneration. This treatment seems to rescue the nerve representation in S1, thus minimizing the stabilization of new representations of adjacent body parts in regions previously responsive to the MN.

## INTRODUCTION

After trauma to the peripheral nervous system, return to full function is seldom achieved, particularly following nerve transection (Lo et al., 2014), or worse, when an entire segment of the nerve is lost. Among other therapeutic strategies, mesenchymal stem cell (MSC) engraftment combined with conduit repair have emerged as promising tools for promoting nerve regeneration (Dezawa et al., 2001; Pereira Lopes et al., 2006; Ribeiro-Resende et al., 2009; Frattini et al., 2012; Oliveira et al., 2013).

The synthetic policaprolactone (PCL) conduit is biodegradable and biocompatible (Den Dunnen et al., 1993; Chang, 2009). Also, this biopolymer is an adequate substrate to support the adhesion of Schwann cells, as well as MSC (Schnell et al., 2007; Chiono et al., 2009; Oliveira et al., 2010; Frattini et al., 2012). MSC secrete several trophic factors, supporting neuritogenesis and neurite growth *in vitro* (Gu et al., 2010) and survival and elongation of the growth cone *in vivo* (Pereira Lopes et al., 2006; Chen et al., 2007; Pan et al., 2007; Ribeiro-Resende et al., 2009). Furthermore, these cells are easily obtained, isolated, and expanded *in vitro*; they have paracrine effects and migratory behavior; and unlike embryonic stem cells, no ethical issues impede their use (Brooke et al., 2007), and the risk of tumorigenesis is negligible. In addition, there is the possibility of an autologous transplant, avoiding tissue-rejection effects.

Recently, our group demonstrated the positive effects of MSC engrafted into a polycaprolactone (PCL) conduit on regeneration after MN transection in mice (Oliveira et al., 2010), but there is still no direct evidence that the regenerated nerve provides functional inputs to the central nervous system (CNS).

It is well known that peripheral nerves of the brachial plexus, like the median, ulnar, and radial nerves, are responsible for carrying information from cutaneous receptors of the forepaw to the CNS. In the rat, Waters et al. (1995) showed that, in S1, the representation of the forepaw is topographically organized; and individual digits correspond to different barrel rows in the forepaw barrel subfield (FBS). However, it is still not clear where in the rat cortex inputs coming from the different brachial plexus nerves are represented. In a functional magnetic resonance study, Cho et al. (2008) attempted to clarify this issue by electrically stimulating individual peripheral nerves from the forepaw; but due to intrinsic limitations of the technique, the representation of inputs carried by individual nerves was inaccurate and anatomical colocalization with barrels was not performed.

The remarkable ability of S1 to reorganize itself in response to peripheral lesions has been widely documented (Kaas et al., 1983; Merzenich et al., 1983b; Jones, 2000; Pearson et al., 2001, 2003; Weng et al., 2011). In primates, MN crush leads to instant modifications of the hand representation that revert, some weeks later, to what was previously mapped as the normal hand representation before the occurrence of the lesion (Wall et al., 1983). However, when the MN is sectioned and repaired by direct suture of the stumps, the cortical representation of the reinnervated skin is abnormal, and reestablishment of the previous cortical map does not occur (Wall et al., 1986).

In this study, we used S1 responsiveness and plasticity as parameters for accessing the functional recovery of a lesioned MN. In order to identity the region of S1 receiving inputs from the MN, all other brachial plexus nerves but the MN were sectioned immediately before electrophysiological mapping. This procedure ensured that cortical responses to forepaw stimulation could only be due to activation of MN fibers. In animals from two additional experimental groups, we removed a segment of the MN and immediately treated this lesion by suturing the stumps to a PCL tube containing or not MSC 10 weeks before mapping S1. Like in the first experimental group, S1 mapping was performed only after acute deprivation of inputs from all brachial plexus nerves with the exception of the MN.

## MATERIALS AND METHODS

### ANIMALS AND EXPERIMENTAL GROUPS

A total of 17 adult male Wistar rats were used in this study. Ten of these animals were submitted to electrophysiological mapping procedures as part of the three different experimental groups described below (see **Table 1**). Another four animals were used only for increasing the sample for the morphological study of the MN. The three remaining animals were used for the sole purpose of harvesting MSC from the bone marrow.

**Table 1:** List of experimental cases and respective number of electrophysiological recording sites in the somatosensory cortex.

Case	Experimental group	Total # of sites	# of sites inside RMN	# of sites sourrounding RMN	# of sites in RMN that changed responses after MN section	# of sites surrounding RMN that changed responses after MN section
11-02	*MN Intact*	73	22	51	–	–
11-03	*MN Intact*	78	20	58	5	3
11-04	*MN Intact*	91	8	85	8	7
11-12	*MN Intact*	64	27	33	3	4
11-05	*PCL-Only*	63	10	53	–	–
11-06	*PCL-Only*	61	10	51	–	–
11-13	*PCL-Only*	40	5	35	–	–
11-09	*PCL+MSC*	60	21	39	–	–
11-10	*PCL+MSC*	56	15	41	–	–
11-11	*PCL+MSC*	53	18	35	–	–

All efforts were devoted to minimize animal suffering and to keep animal welfare along the period of experiment. Although the transection followed by conduit repair could lead to pain, in this study, we did not observe any pain behavior, such as stress or autotomy. All experiments were approved by the Ethics Committee on the Use of Experimental Animals of the Federal University of Rio de Janeiro (protocol # IBCCF 112) in accordance to Brazilian Law and the National Institutes of Health guidelines.

Our first experimental group, called *MN Intact* (*n* = 4 animals), served as a control for the other two experimental groups. In the *MN Intact* group, we mapped the cortical representation of inputs carried by the MN after its functional isolation by sectioning all other brachial plexus nerves immediately before the mapping session. In this experimental group, the same cortical region was mapped a second time, during the same experimental session, right after sectioning the MN itself. This allowed us to investigate S1 immediate plasticity after deprivation of the MN inputs.

In the second (*PCL-Only*) and third (*PCL+MSC*) experimental groups, animals underwent resection of a segment of the MN, 10 weeks before mapping S1. This lesion was immediately followed by microsurgical reconstruction with a PCL conduit implantation filled with Dulbecco’s Modified Eagle Medium (DMEM, in the *PCL-Only* group, *n* = 3) or with MSC in DMEM (in the *PCL+MSC* group, *n* = 3). Mapping procedures were performed under the same conditions as in *MN Intact* group. Thus, in order to identify, in S1, the representation of the regenerated MN, all brachial plexus nerves, with the exception of the treated MN, were sectioned immediately before mapping S1 in animals from *PCL-Only* and *PCL+MSC* groups. Mapping data from individual experiments in the three experimental groups were then compared.

### BM-MSC CULTURE

Bone marrow was flushed with Hank’s buffered saline solution (HBSS) from femurs and tibias of donor rats and centrifuged at 2000 rpm for 5 min. Cells were seeded into 25 cm^2^ culture flasks at a density of 5 × 10^7^ containing DMEM supplemented with 20% fetal bovine serum and 100 U/mL penicillin. Cells were incubated at 37°C in 5% CO_2_ for 3 days. Next, the non-adherent cells were removed from the flasks and adherent cells were washed once with HBSS and fed with supplemented medium. These adherent cells were cultured and passaged three times until the final use. The culture medium was replaced three times per week. Before transplantation, cells were re-suspended at a density of 10^6^ cells in 4 μL, and then injected into the PCL conduit at the time of surgery. After surgery, a sample of the remaining cells was plated overnight, to assess cell viability. Cells were maintained in culture for 24 h and their morphology analyzed.

### SURGICAL PROCEDURES

For animals belonging to the *PCL-Only* and *PCL+MSC* groups, a two-stage experiment was performed. Initially, animals were profoundly anesthetized with ketamine (100 mg/kg) and xylazine (15 mg/kg). After being exposed from the axilla to the cubital fossa, the right MN was transected at the level of the middle of the *brachialis* muscle. A 4-mm long segment of the nerve was then resected. The proximal stump was sutured through the epineurium to a 10-mm length PCL conduit (Tonda-Turo et al., 2011) using a 10.0 micronylon suture (MICROSUTURE, BRAZIL). MSC in DMEM (2 × 10^6^/4 μL, for animals on the the *PCL+MSC* group) or DMEM only (for animals from the *PCL-Only* group) were injected into the conduit with a Hamilton micro syringe. Finally, the distal stump was sutured to the other end of the PCL conduit, leaving an 8-mm gap between the nerve stumps.

After 10 weeks, these animals underwent a second surgical intervention in which all other brachial plexus nerves with the exception of the MN were sectioned, and the cortical mantle exposed for electrophysiological recording (see below). Animals from the *MN Intact* group were only submitted to the second (mapping) experimental stage.

### ELECTROPHYSIOLOGY

Animals were initially anesthetized with intramuscular injections of ketamine hydrochloride (100 mg/kg) and xylazine (15 mg/kg), and then atropine (0.2 mg/kg) and dexamethasone (0.6 mg/kg) were administered. Surgical levels of anesthesia were maintained with supplemental doses of ketamine and xylazine delivered intramuscularly. Throughout the experiment, heart and respiration rates were monitored and body temperature was maintained at 37°C.

After adequate anesthetic level was obtained, brachial plexus nerves from the right side were exposed and then transected, with the exception of the MN. Then, the animal was positioned in a stereotaxic holder, the skin was cut, the temporalis muscle was retracted, and a craniotomy was made over the left parietal cortex. The exposed cortical surface was covered with silicone oil (MERCK CHEMICALS, USA). A magnified photograph of the exposed cortical surface was taken to mark the sites where electrode penetrations were placed in relation to superficial blood vessels.

S1 multiunit recordings were performed under standard mapping conditions as previously described in other studies of our group (Anomal et al., 2011). Basically, a tungsten microelectrode (1–5 MW at 100 Hz; A-M Systems, Sequim, WA, USA) was inserted perpendicular to the cortical surface at about 700 μm deep into the cortical tissue. Multiunit activity was amplified, filtered, and monitored through an audio speaker while the contralateral body surface was stimulated by light to moderate displacement of the skin with fine-tipped probes. Light to moderate taps, digit and limb manipulation, and light pressure were used to stimulate deep receptors of the muscles, joints, and skin. Multiunit cortical responses to light cutaneous stimuli were classified as good, weak or absent. Good cutaneous responses consisted in clear multiunit activity identified as sudden and repetitive increases in the intensity of the background noise coincident with the repetitive stimulation of the surface of the skin.

The somatosensory representation of the forepaw and its surroundings were carefully mapped by inserting the electrode at different cortical surface positions separated in the *x–y* plane by increments of 300 μm. The receptive field of each recording site and the quality of the response were documented in drawings of the body surface. Recording sites with neurons unresponsive to any type of somatosensory stimulus or with neurons presenting weak cutaneous responses or exclusive responses to deep somatosensory stimuli were also documented. After mapping the representation of the forepaw and its surroundings in animals from the *MN Intact* group, the MN itself was cut to deprive the brain from all information originating in the forepaw. Then, the same sites were remapped and acute modifications of multi unit receptive fields previously located at the forepaw were documented (see below).

After the mapping session, small DY crystals (Sigma, St. Louis, MO, USA) were placed at 3 or 4 cortical sites to allow superimposition of the mapping data with histology for cytochrome oxidase (CO). The position of the DY crystals relative to mapping sites was marked in the photograph of the cortical surface.

### HISTOLOGICAL PROCESSING

By the end of the mapping experiment, a supplemental dose of anesthesia was given. After a deep anesthesia level was achieved, the animal was transcardially perfused with 0.9% saline (50 mL/animal), followed by a fixative solution (4% paraformaldehyde in 0.1 M phosphate buffer, pH 7.4). After carefully removing the brain from the skull, the left cerebral cortex was carefully separated from subcortical structures and flattened overnight by placing it between two lightly weighted microscope slides in phosphate buffer with 30% sucrose. Serial tangential frozen sections of the cortex were made on a cryostat (Leica CM 1850, USA) at a thickness of 30 μm. Alternate histological sections were processed for CO (Wong-Riley and Welt, 1980), or left unprocessed for visualization by fluorescence microscopy. Sections were placed on gelatin-coated slides, dehydrated, and finally mounted with Entellan (MERK, USA).

Following perfusion, a 2-mm long segment of MN was harvested from the middle portion of the PCL conduit. This nerve segment was fixed by immersion in 2.5% glutaraldehyde diluted in 0.1 M cacodylate buffer (pH 7.4) for 2 h, washed in 0.1 M phosphate buffer (pH 7.4) followed by 0.1 M cacodylate buffer (pH 7.4), and post-fixed for 2 h in 1% osmium tetroxide containing 0.8% potassium ferrocyanide and 5 nM calcium chloride in 0.1 M cacodylate buffer (pH 7.4). The nerve segment was washed in 0.1 M cacodylate buffer (pH 7.4) and distilled water and stained in 1% uranyl acetate overnight, dehydrated in graded acetone, infiltrated with Poly/Bed 812 resin (Polysciences Inc., Washington, PA, USA) and polymerized at 60°C for 48 h. Semithin cross sections (500 nm) were obtained and stained with toluidine blue for light microscopy analysis. Ultrathin cross sections (70 nm) were collected on copper grids and contrasted in uranyl acetate and lead citrate. Microscopy analysis and documentation were carried out using a Zeiss 900 transmission electron microscope operated at 80 Kv (Zeiss EM 900).

### DATA ANALYSIS

The photograph of the cortical surface with the location of mapping sites and DY crystals was superimposed on drawings of CO-stained tissue sections made in a Zeiss Axioplan-2 microscope equipped with a color digital camera (1600 × 1200, 3/4” chip, 36bit, MBF) and a motorized stage (Mac5000 LUDL) controlled by Neurolucida software (MBFBiosciences, Inc.) running on a Dell workstation. Drawings made in Neurolucida contained contours of individual barrels and barrel subfields, and position of the DY crystals as identified by fluorescence microscopic analysis. Using the DY crystals as fiducial markers, the superimposition between the photograph and the drawing of the histological section allowed the correlation between mapping sites and CO barrels. Barrel fields could then be used as anatomical markers of the representation of different body parts (Santiago et al., 2007), against which mapping results could be compared.

Somatosensory maps were drawn using standard procedures. Basically, borders between different sensory representations were marked at an equal distance between two recording sites where neuronal responses to stimulation of different body parts were obtained. In our preparation, the cortical representation of inputs carried by the MN in S1 comprised the area containing sites where good responses to superficial cutaneous stimulation of the forepaw were obtained. Before measuring the area of the representation of the MN in S1 (RMN) obtained in different mapping experiments, we first chose a composite drawing from one experiment as reference, and then adjusted the size of the drawings from the other experiments, adjusting their sizes until the superimposition of the barrel rows of the postero medial barrel subfield (the large conspicuous barrels that represent the vibrissae) of all drawings accurately matched by superimposition. This procedure minimized variations due to differential tissue retraction that could be occurring in different experiments.

We performed qualitative and morphometric analysis of the 2-mm long segment of the MN extracted from the PCL conduit in animals of the *PCL-Only* and *PCL+MSC* experimental groups. We acquired images from these MN segments in an electron microscope at a magnification of 7000× for qualitative analysis.

For the quantitative analysis, we photographed the whole semithin cross section of the nerve under light microscopy using a 100× oil objective and the Neurolucida Virtual Slice Module (MBF, Inc.). Then, we counted the total number of myelinated nerve fibers in the resulting microphotograph. Also, five systematic fields in each nerve cross section were sampled for measurement of fiber and axon diameters, g-ratio (axon diameter divided by fiber diameter) as well as myelin thickness. All measurements were made using Image J Software.

### STATISTICAL ANALYSIS

For MN histomorphometric analysis, paired inferences were made by Student’s *t*-test. The confidence interval was 95%, with an accepted alpha value of 5% (*p* < 0.05). The analyses were carried out using the Prism Graph Pad 4.0 statistical software.

## RESULTS

We designed our experiments to study both the repaired MN morphology and the representation of MN inputs in S1. In order to evaluate differences between the cortical representation of intact and regenerated nerves, and to further characterize the impact of MSC engraftment into the PCL tube, three experimental groups were studied: *MN Intact* (*n* = 4), *PCL-Only* (*n* = 3), and *PCL+MSC* (*n* = 3). In all three experimental groups, all brachial plexus nerves, with the exception of the MN, were acutely sectioned immediately before mapping procedures. Under this experimental condition, we identified the cortical representation of median nerve (MN) inputs in S1, the RMN, as the region of S1 containing neurons responsive to light cutaneous stimulation of the forepaw. We mapped the RMN in 10 experiments, comprising a total of 639 mapping sites. **Table 1** lists all mapping cases and the number of recording sites inside and surrounding the RMN in each individual mapping experiment reported in this study. An enlargement of the RMN was correlated with the treatment applied to the peripheral nerve, namely a higher number of myelinated fibers (MFs) in the appropriate g-ratio range was obtained in the *PCL+MSC* group as compared to the *PCL-Only* group.

### MSC ENGRAFTED IN PCL TUBES ENHANCE MN REGENERATION

**FIGURE 1:**
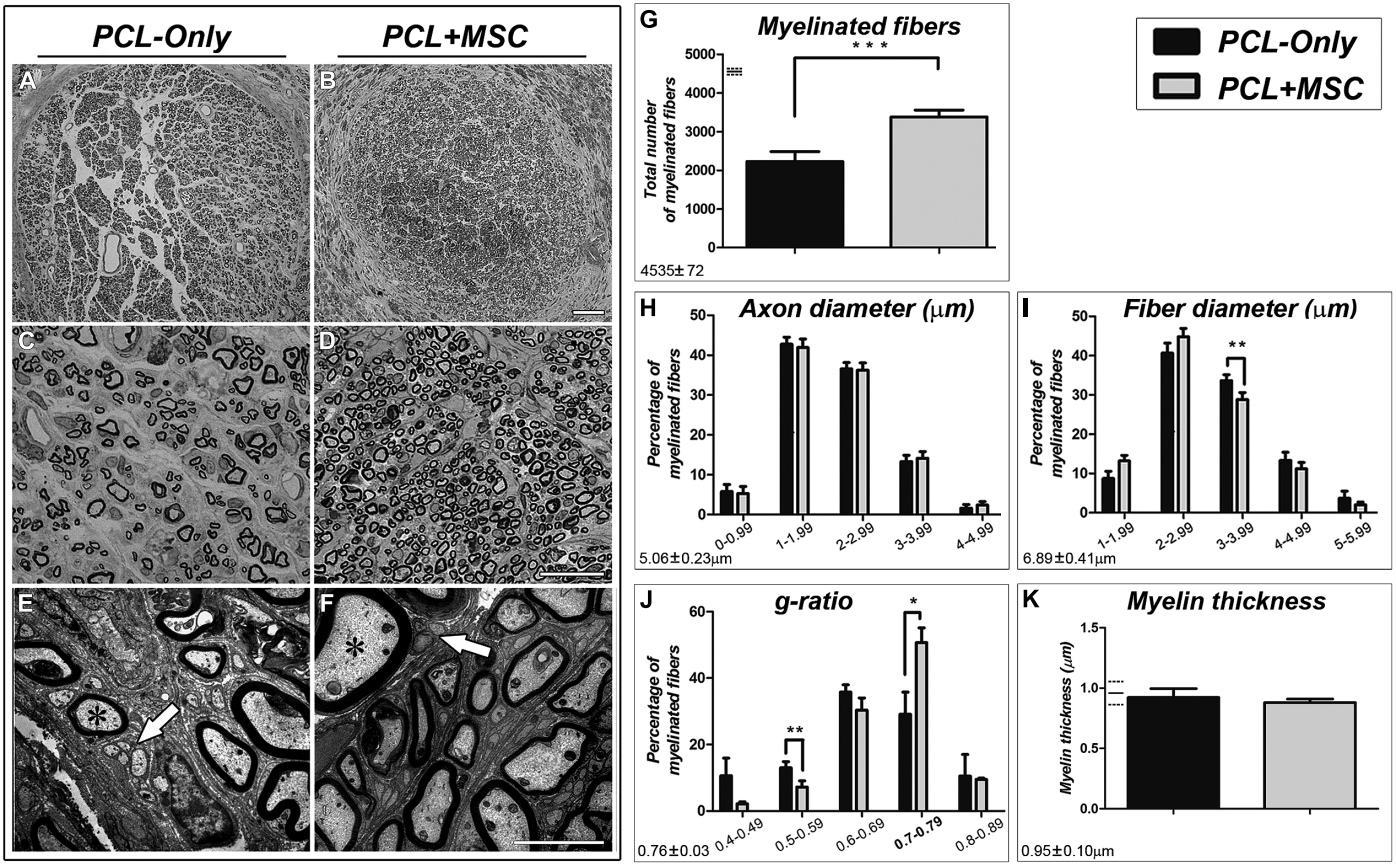
**Morphological analysis of the regenerated MN. (A**,**B)** Low-magnification micrographs of toluidine blue stained semi-thin cross sections of the MN regenerated through the PCL tube in *PCL-Only* **(A)** and *PCL+MSC* **(B)** groups. Scale bar: 50 μm. **(C,D)** Higher magnifications of **(A,B)**, exhibiting clusters of regenerating fibers (fascicles). Scale bar: 20 μm. **(E,F)** Electron micrographs of ultrathin cross sections of the MN, displaying myelinated (asterisks) and non-myelinated (arrows) nerve fibers. Scale bar: 2 μm. **(G–K)** Histomorphometric analysis of the regenerated MN. Values described for the normal nerve by Muratori et al. (2012) for each parameter analyzed are depicted in the bottom left corner and at the left of the graphs (in **G,K**) as horizontal lines representing the mean (continuous line) ±standard deviation (dashed lines). **(G)** The total number of myelinated fibers (MFs) in the *PCL+MSC* group (gray bar) is higher than in the *PCL-Only* group (black bar; *p* < 0.001). **(H)** Percentage of MFs in different ranges of axon diameters. **(I)** Percentage of MFs in different fiber diameter ranges. Fiber diameter corresponds to the diameter of the axon plus the thickness of the myelin sheath. In the *PCL-Only* group, there was a higher percentage of MFs in the 3.00–3.99 m range than in the *PCL+MSC* group (*p* < 0.01). **(J)** G-ratio analysis showed a higher percentage of MFs in the *PCL-Only* group than in the *PCL+MSC* group in the 0.70–0.79 range (*p* < 0.05), which is similar to values found in the normal rat MN (Muratori et al., 2012). **(K)** The thickness of the myelin sheath is equivalent when all fibers of the two groups are compared. ^∗^*p* < 0.05, ^∗∗^ *p* < 0.01, ^∗∗∗^*p* < 0.001.

After mapping the RMN in *PCL-Only* and *PCL+MSC* cases (see below), the repaired MN of each experimental subject was transversally sectioned at the level of the PCL conduit and prepared for histological analysis. The structure of the nerve was evaluated and the number of fibers was counted (**Figure 1**). Regenerated nerves from the *PCL+MSC* group (**Figures 1B,D**) displayed a more organized cytoarchitecture than those of the *PCL-Only* group (**Figures 1A,C**). In nerves from the *PCL+MSC* group, there were clusters of regenerating fibers surrounded by processes of perineurium-like cells forming fascicle-like clusters that were not so evident in nerves from the *PCL-Only* group. In the *PCL-Only* group, these clusters were more dispersed and presented smaller sizes than those observed in the *PCL+MSC* group (compare **Figures 1C,D**). Electron microscopy revealed that regenerated nerves from both *PCL-Only* and *PCL+MSC* groups exhibited myelinated nerve fibers with well-defined axoplasm and well-organized myelin sheaths as well as many small non-MFs (**Figures 1E,F**).

Morphometric analysis of the regenerated MN showed a significantly higher number of MFs in nerves from the *PCL+MSC* group (3,383 ± 390.3; *n* = 5) than in the *PCL-Only* group (2,226 ± 575.1; *n* = 5; *p* < 0.001; **Figure 1G**). There was no difference between the *PCL-Only* and *PCL+MSC* groups when the percentage of MFs was compared in the different ranges of axon diameter (range 0–0.99: 5.8 ± 4%, 5.3 ± 4%; range 1.00–1.99: 43 ± 4%, 42 ± 5%; range 2.00–2.99: 37 ± 3.6%, 36 ± 4%; range 3.00–3.99: 13 ± 3.5%, 14 ± 3.7%; range 4.00–4.99: 1.6 ± 2%, 2.4 ± 2%; respectively, *n* = 5 per group, **Figure 1H**). However, the analysis of the fiber diameter (i.e., the diameter of the axon plus the thickness of the myelin sheath) in different diameter ranges showed that the *PCL-Only* group presented a higher percentage of MFs in the 3.0–3.99 μm range of fiber diameter than the *PCL+MSC* group (range 1.0–1.99: 8.7 ± 4%, 13 ± 3%; range 2–2.99: 40.6 ± 5.7%, 44.8 ± 4.8%; range 3–3.99: 33.7 ± 3.4%, 28.8 ± 4.1%, *p* < 0.01; range 4–4.99: 13.3 ± 4.7%, 11.2 ± 3.6%; range 5–5.99: 3.7 ± 4%, 2 ± 1.5%; respectively, *n* = 5 per group, **Figure 1I**).

The comparison of the g-ratio (i.e., axon diameter divided by fiber diameter) demonstrated that both *PCL-Only* and *PCL+MSC* groups presented a Gaussian distribution pattern as expected (**Figure 1J**). However, the *PCL+MSC* group showed a tendency toward higher g-ratios than the *PCL-Only* group. Additionally, the *PCL+MSC* group presented a higher frequency of MFs in the 0.70–0.79 range than the *PCL-Only* group (respectively 50.7 ± 9.9% and 29.1 ± 14.9%, *p* < 0.05), which corresponds to the optimal g-ratio in the MN of adult rats (Muratori et al., 2012). The significantly lower g-ratio frequency for the *PCL+MSC* than for the *PCL-Only* group in the lower range of 0.50–0.59 (respectively, 7.2 ± 4.1 and 13 ± 4.1, for *PCL+MSC* and *PCL-Only* respectively, *p* < 0.01) confirmed a bias of the *PCL+MSC* group toward the highest g-ratios (**Figure 1J**). Frequency of g-ratios in the other ranges was not significantly different when *PCL+MSC* and *PCL-Only* groups were compared (respectively, in the 0.40–0.49 range: 2.1 ± 1.3%, 10.6 ± 12%; in the 0.60–0.69 range: 30.4 ± 8.2%, 35.8 ± 4.9%; in the 0.80–0.89 range: 9.5 ± 0.8%, 10.5 ± 14.6%, *n* = 5 per group).

No difference in myelin thickness was observed between *PCL-Only* and *PCL+MSC* groups when all fibers of the sample were compared together (0.9 ± 0.2 m, 0.9 ± 0.1 m; respectively, *n* = 5 per group).

### MN REGENERATION THROUGH A PCL TUBE REESTABLISHES ELECTROPHYSIOLOGICAL RESPONSES IN RAT S1

One of the main objectives of this study was to investigate whether the tubulization of a previously transected MN would reinnervate the skin, providing cutaneous inputs to S1, 10 weeks after the lesion. We mapped cutaneous inputs from MN fibers in the FBS region of area S1 and its surroundings, by acutely transecting all other brachial plexus nerves in an anesthetized preparation. In all three experimental groups, neurons in the RMN could be activated by gentle stimulation of different parts of the forepaw, including most of the glabrous and dorsal (hairy) forepaw, with the exception of the glabrous and hairy sides of the fifth digit.

**FIGURE 2:**
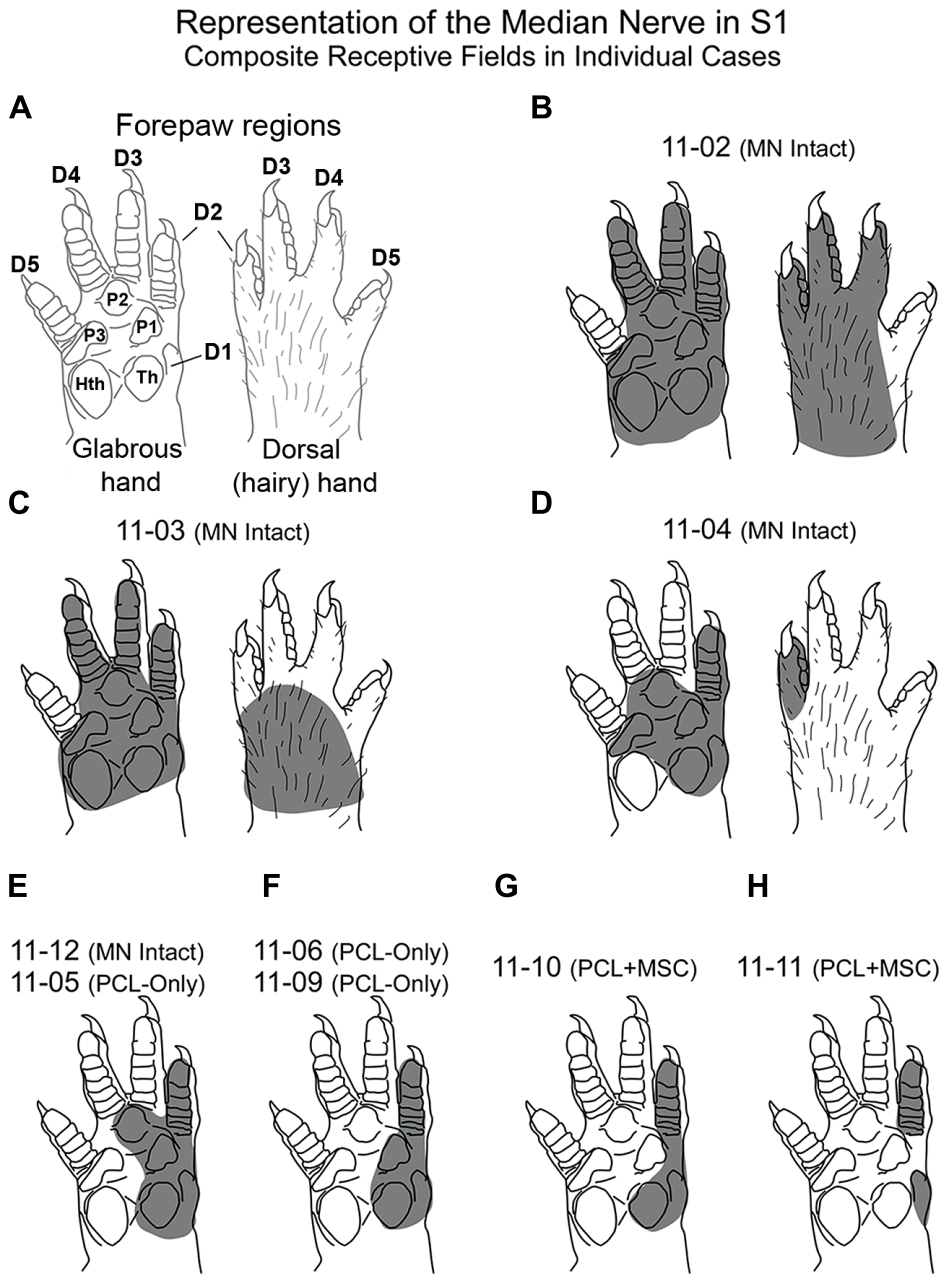
**Composite receptive fields (shaded area) in nine different mapping experiments from the three experimental groups.** These composite receptive fields can be interpreted as the part of the skin innervated by the isolated MN. (Cortical maps from these experiments are illustrated in **Figures 4–7**). **(A)** Schematics showing the different regions of the rat forepaw: D1–D5 and palmar pads, including the Th and HTh pads, and the pads close to the digits (P1, P2, P3). **(B**–**H)** Composite receptive fields varied in individual cases. Cases presenting the same composite RF were illustrated together **(E,F)**. In cases from the *MN intact* group (in **B–E**), the RMN always included the representation of Th, P1, and P2, plus the glabrous skin of D1 and D2. In *PCL-Only* **(E,F)** and *PCL+MSC* **(G,H)** experiments, composite RFs mapped in the RMN were restricted to smaller areas of the glabrous skin of the forepaw located close to and including D1 and D2. Quantitative data for each experiment (except for cases 11-05 and 11-06) are shown in **Figure 3**.

**Figure 2** shows composite receptive fields obtained in individual mapping experiments from all three experimental groups. In **Figure 2**, the shaded area in each drawing of the forepaw corresponds to the region in which light cutaneous stimulation was able to evoke clear cortical responses in S1 neurons, considering all recording sites of a given experiment. These composite receptive fields can be interpreted as the part of the skin innervated by the isolated MN. We observed some variability in the size of composite receptive fields obtained in individual cases, especially when *MN Intact* animals were compared with those in which MNs had been transected (**Figure 2**). However, presence of neuronal responses to cutaneous stimulation of the forepaw in animals from the *PCL-Only* and *PCL+MSC* groups demonstrated that the previously lesioned MN was able to regenerate and carry inputs capable of activating cortical neurons in S1, at least in our experimental conditions.

**FIGURE 3:**
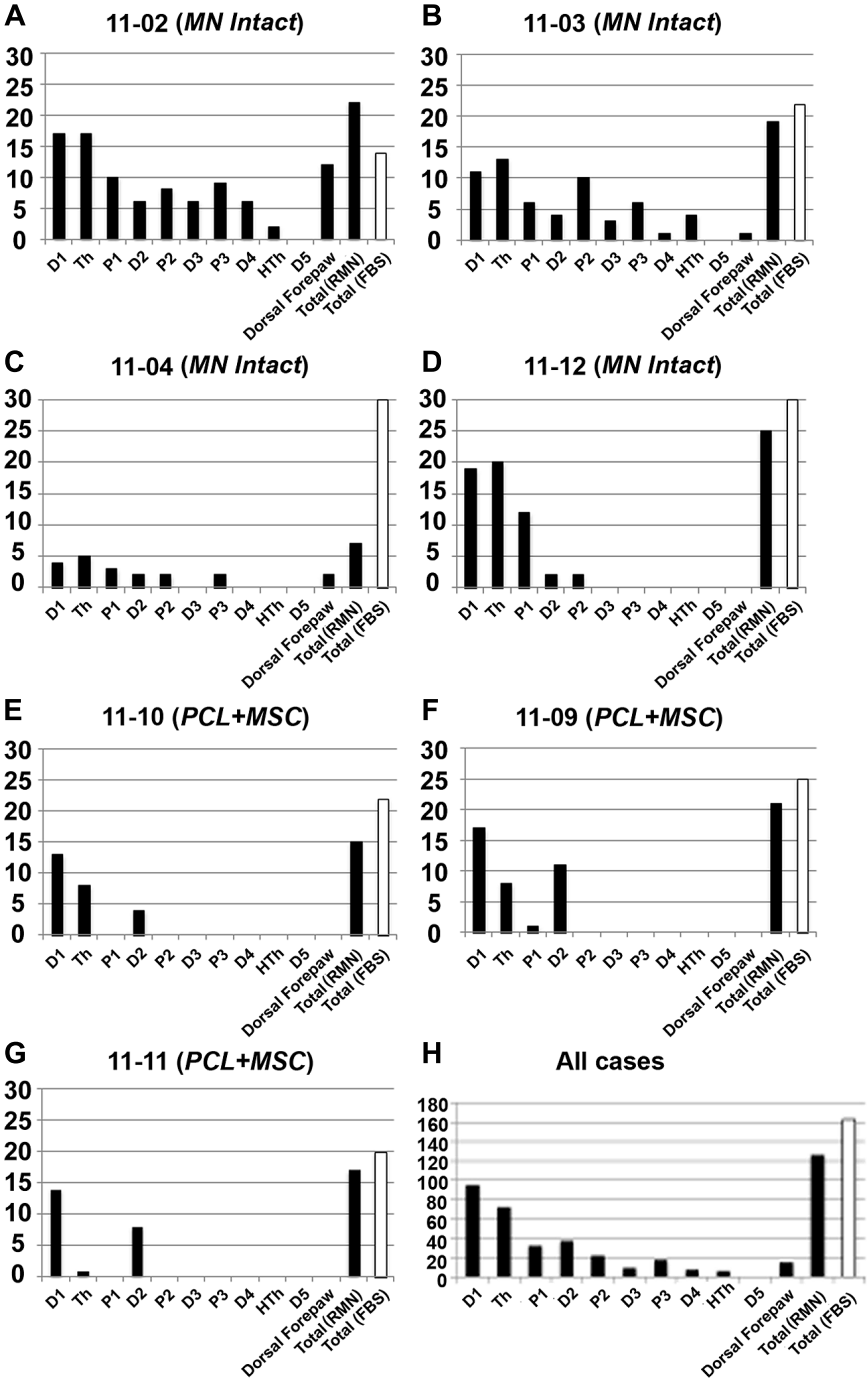
**Quantification of the representation of different parts of the forepaw in the RMN.** The number of recording sites with neurons responsive to cutaneous stimulation of different forepaw regions is illustrated in seven experiments (*y-*axis in **A–G**). The summation of all seven experiments is shown in **(H)**. The forepaw was subdivided in the 11 regions depicted in **Figure 2A**: D1–D5; Th, HTh, P1, P2, and P3; plus dorsal forepaw. Only sites containing neurons responsive to superficial cutaneous stimulation of the forepaw (thus part of the RMN) were quantified (black bars). For comparison, the number of sites inside the FBS, whether containing neurons responsive to cutaneous stimulation or not, was also illustrated (white bars). “Total (RMN)” refers to the number of sites inside the RMN. Note that a single multiunit receptive field mapped in a given recording site could encompass more than one forepaw region. Thus, this site would be represented in more than one bar in the graph, since each bar corresponds to a single forepaw region. RMN mapped in the *MN Intact* group seemed to encompass broader regions of the forepaw than in the *PCL+MSC* group. D1 was the most represented parts of the forepaw in all experiments, being closely followed by Th. Composite receptive fields of these experiments are represented in **Figure 2**. Respective electrophysiological maps are represented in **Figures 4**–**7**.

In our preparation, the set of cortical recording sites containing neurons clearly responsive to superficial stimulation of the forepaw was defined as the RMN. In the RMN, the representation of some parts of the forepaw, like the first digit (D1) and of the thenar pad (Th) were represented in a higher number of recording sites than other parts of the forepaw like the other digits and dorsal hand. **Figure 3** shows this quantification in individual mapping experiments of the *MN intact* and from *PCL+MSC* groups (**Figures 3A-G**). In the *MN intact* group (*n*= 4 experiments), most of the 77 recording sites in the RMN (**Table 1**) presented neurons activated by stimulation of the glabrous skin of D1 (70% of the sites) and Th (75% of the sites), being followed by the digit one and digit two pads (P1: 42%, P2: 30%; respectively). Responses to stimulation of other parts of the glabrous paw, such as the digit 2 (D2: 19%) and the third digit palm pad (P3: 23%), were also observed. Digit 4 (D4: 10%), hipothenar pad (HTh: 8%), and digit 5 (D5: 0%) were the least represented parts of the glabrous forepaw. Cutaneous stimulation of regions of the dorsal (hairy) forepaw, with the exception of D5, evoked responses in 21% of the recording sites performed in the *MN Intact* group. After nerve transection and treatment, the region of the forepaw in which cortical neuron responses were elicited was more restricted than in the *MN Intact* group (**Figures 2E–H**). More specifically, in the *PCL+MSC* group (*n* = 3 experiments comprising a total of 54 sites in the RMN, **Table 1**), neuronal responses were obtained only after stimulation of D1 (83% of the sites), D2 (43%), Th (32%), and P1 (2%; **Figures 3E–-G**). This result in the *PCL+MSC* group probably reflects the natural bias of the unlesioned MN to innervate the radial side of the glabrous hand, with predominance of D1 and Th (as seen in **Figure 3H** for the totality of recording sites illustrated in **Figure 3**).

### TREATMENT WITH MSC ENLARGES THE CORTICAL REPRESENTATION OF A PREVIOUSLY TRANSECTED MN

**FIGURE 4:**
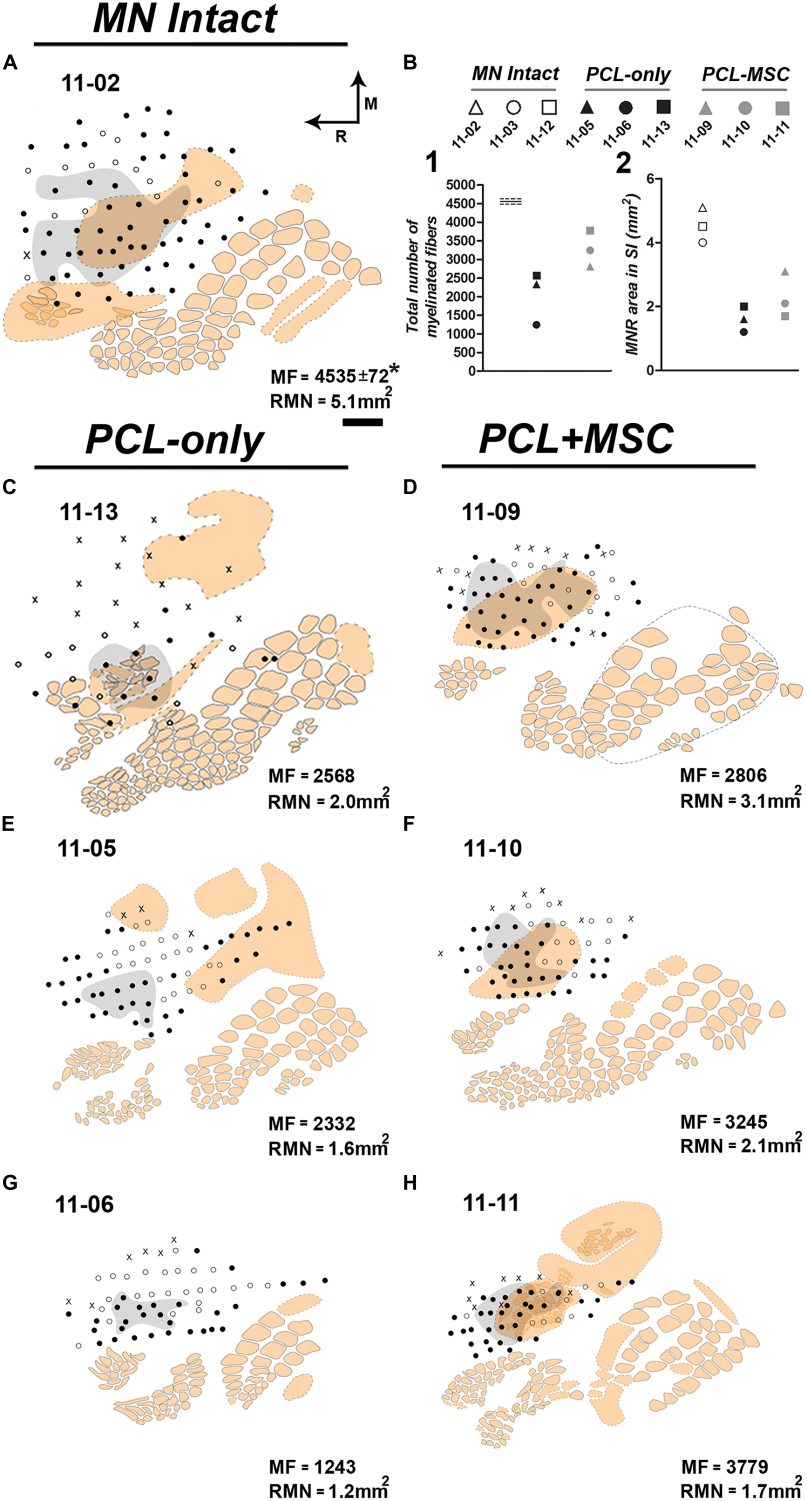
**Examples of the region covered by the RMN (gray zone) mapped in individual experiments from different experimental groups and respective quantitative data.** The RMN corresponded to the region of S1 containing neurons responsive to superficial cutaneous stimulation of the forepaw after all brachial plexus nerves were cut, except for the MN. The cortical region mapped comprised the FBS and its surroundings. **(A)** Case 11-02 from the *MN Intact* group. In this experiment, the RMN occupies 5.1 mm^2^ (as depicted in the inferior right corner). MF in the normal (not previously cut) MN was obtained by Ronchi et al. (2009). **(B)** Quantitative data from individual experiments of the *MN Intact* (white labels), *PCL-Only* (black labels), and *PCL+MSC* (gray labels) groups. **Top:** labels used in the graphs below representing individual experiments. **Left:** number of MFs in the MNs that regrew through a PCL tube. For reference, MF in the normal nerve obtained by Ronchi et al. (2009) is represented as horizontal lines on the left of the graph (continuous line: mean; dashed lines: standard deviations). **Right:** size of the area of the RMN in nine experiments. **(C,E,G)** Individual experiments from the *PCL-Only* experimental group. Ten weeks before mapping procedures, animals in this group underwent resection of a 4-mm long segment of the MN, followed by immediate repair with microsurgical implantation of a PCL tube through which the MN could regrow from the proximal to the distal stump, and then reinnervate the forepaw. After mapping procedures, the repaired MN was transversally cut at the level of the PCL tube, submitted to conventional histological processing, and MF was counted in semi-thin sections under light microscopy. MF and the area occupied by the RMN are both depicted in the lower right corner of each experiment. **(D,F,H)** *PCL+MSC* group. This group underwent the same procedures adopted in the *PCL-Only* group plus the injection of MSC into the PCL tube before suturing the distal stump of the MN to the tube. RMN area in *MN Intact* group showed higher values compared with MN repaired groups (11-03: 4.0 mm^2^; 11-12: 4.5 mm^2^; and 11-02: 5.1 mm^2^). *PCL+MSC* group presented larger RMN areas and higher MFs (1.7 mm^2^, 3779; 2.1 mm^2^, 3245; 3.1 mm^2^, 2806; in **D,F,H**, respectively) than the PCL-Only group (1.2 mm^2^, 1243; 1.6 mm^2^, 2332; 2.0 mm^2^, 2568; in **C,E,G**, respectively). Although both MF and RMN size were higher in the *PCL+MSC* group than in the *PCL-Only* group, a higher number of MF does not necessarily explain a larger RMN. For example, when cases of the *PCL+MSC* group are compared with each other. In **A,C–H**, beige contours represent individual barrels (continuous contours) or regions of intense cytochrome oxidase reactivity in which individual barrels were not clear or not detected (dashed contours). The forepaw barrel subfield (FBS) corresponds to the dashed beige contour in which most of the recording sites (dots) are located. Note that, except for cases 11-05 **(E)** and 11-06 **(G)**, most of the FBS was activated by MN inputs. Filled dots: recording sites with clear responses to light cutaneous stimulation (Filled dots outside the grey area correspond to sites where neurons were responsive to superficial cutaneous stimulation of other body parts not including the forepaw). Empty dots: sites responsive to deep stimulation and/or weakly responsive to superficial stimulation. X: sites not responsive to somatosensory stimulation.

The area mapped as the RMN was measured in individual cases revealing that the extent of the RMN varied somewhat in different experiments. As expected, in *MN Intact* animals the RMNs were the largest ones, ranging from 4.0 to 5.1 mm^2^ (**Figures 4A,B**). In all cases of *PCL-Only* and *PCL+MSC* groups, we recorded neuronal responses to cutaneous stimulation of the glabrous skin conveyed by the regenerated MN (**Figure 4**). In the *PCL-Only* group, the RMN ranged from 1.2 to 2.0 mm^2^, corresponding to around 36% of that measured in the *MN Intact* group. In contrast, extension of the RMN in the *PCL+MSC* group corresponded to around 50% of that measured in the *MN Intact* group, ranging from 1.7 to 3.1 mm^2^. This represented a 43% increase in comparison with the *PCL-Only* group. Animals with higher numbers of MFs in the MN generally presented larger RMNs, especially when animals from the *PCL+MSC* group were compared with those of the *PCL-Only* group (**Figure 4B**). This trend was also observed in individual cases of the *PCL-Only* group (**Figures 4C,E,G**), but unexpectedly not when individual cases of the *PCL+MSC* group were compared with each other (**Figures 4D,F,H**).

### INPUTS CARRIED BY THE UNLESIONED MN CAN ACTIVATE MOST OF THE FBS BUT ARE NOT TOPOGRAPHICALLY ORGANIZED

**FIGURE 5:**
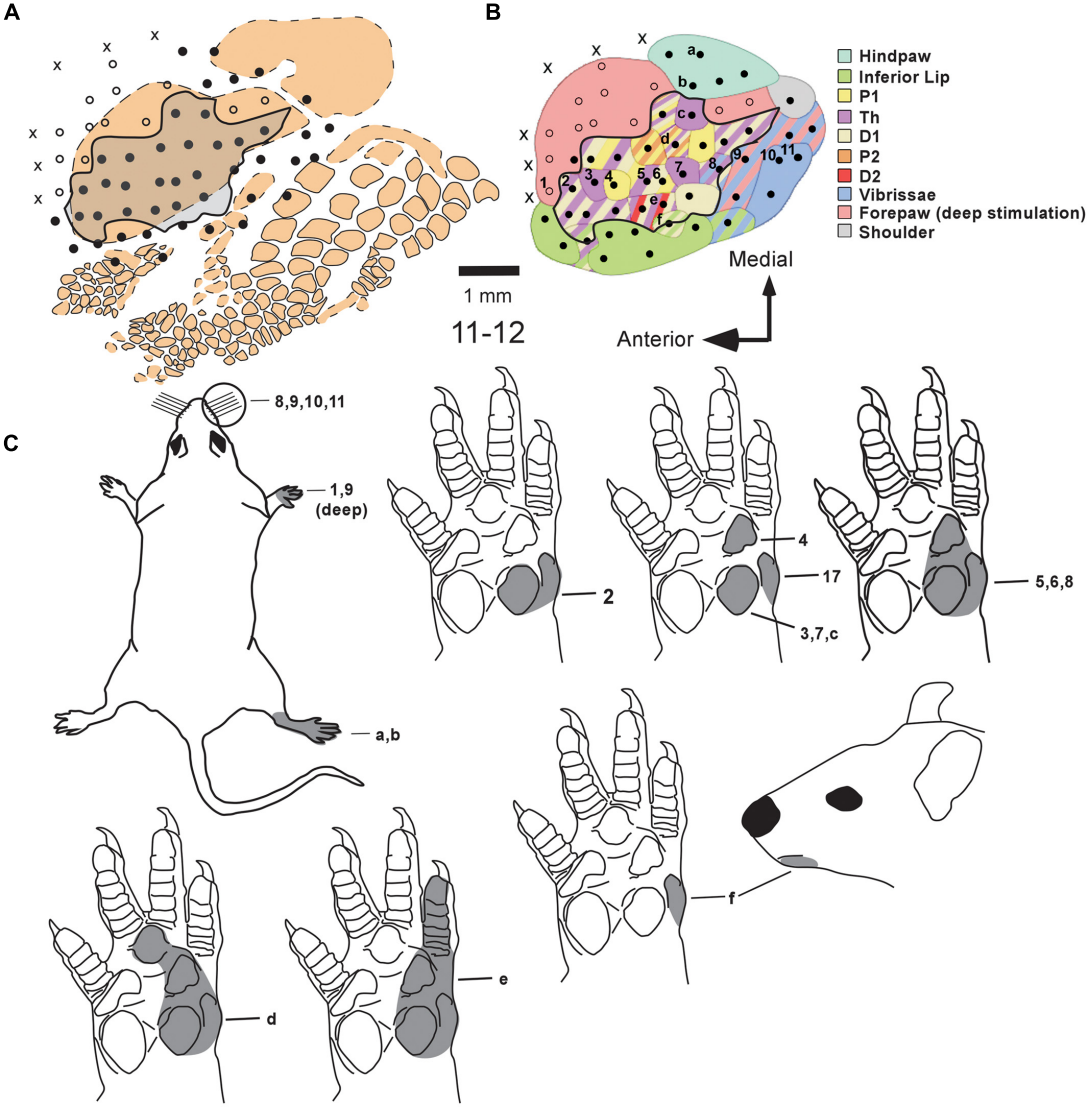
**RMN and barrel fields in case 11-12 (*MN Intact* group).(A)** Schematic representation of the barrel cortex as evidenced by cytochrome oxidase histochemistry obtained in this case and the location of mapping sites. Conventions for representing somatosensory maps and barrel fields are the same as adopted in **Figure 4**. **(B)** Somatosensory map illustrating the representation of different body parts with different colors, as depicted by the legend on the right. Two rows of sites (“1” to “11” and “a” to “f”) provide examples of progressions along the antero-to-posterior and medial-to-lateral axis, respectively. The corresponding multiunit receptive fields of each of these sites are depicted in **(C)** with the same numbers or letters.

We additionally characterized the topography of the RMN in the *MN intact* group. Unexpectedly, we were not able to find a clear topographic organization of the representation of different parts of the forepaw in the RMN. For example, in case 11-12 (**Figure 5**), most of the RMN seemed dominated by the representation of D1, Th, and P1. In some sites neurons responded to stimulation of only one of these three forepaw subregions while in other sites they responded to two of them or to all three, with no apparent topographic order (see row of sites #2 to 9, in **Figure 5B**). Besides, the location and size of the representation of different regions of the forepaw in the RMN varied in different experiments. For instance, in case 11-02, we observed a large representation of the dorsal (hairy) forepaw in the center of the RMN that was not observed in other cases (see quantification in **Figure 3**).

**FIGURE 6:**
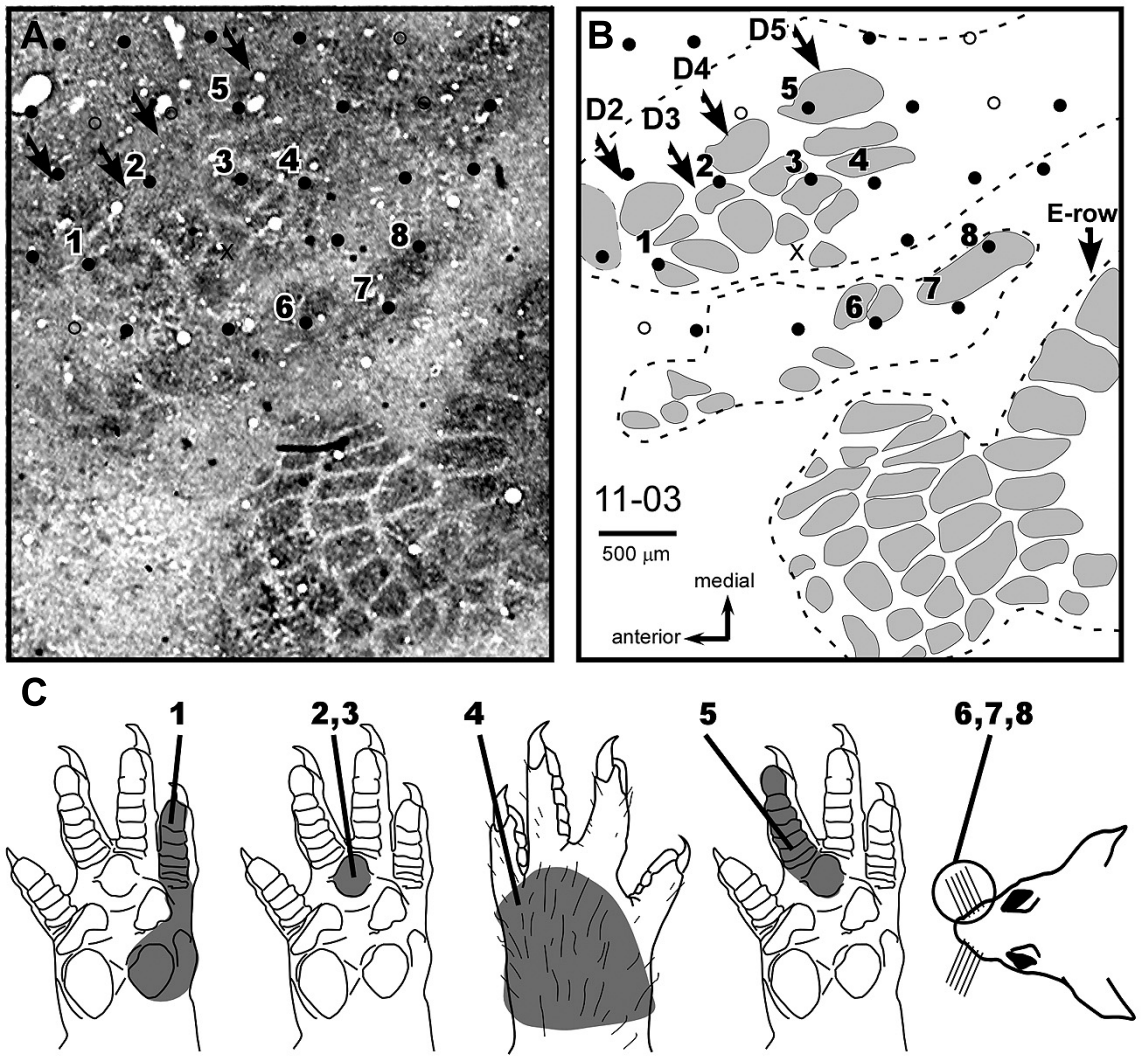
**Neuronal responses relayed by the MN to individual barrels of the FBS.** Case 11-03 (*MN Intact*). **(A)** Composite photomicrograph of two adjacent tangential sections processed for cytochrome oxidase and location of some of the recording sites studied in this experiment. In this preparation, one can identify individual barrels located in the FBS, in the superior and inferior lip barrel fields, and in part of the E-row vibrissae representation of the postero medial barrel subfield (large barrels at the right side of the figure, pointed by an arrow in **B**). Arrows point to FBS barrel rows. **(B)** Schematic drawing corresponding to the photomicrograph in **(A)**. Dashed lines contour three different barrel fields. The top contour corresponds to the FBS. Individual barrels are in gray. The representation of D2–D5 in normal (non-deafferented) rats (as demonstrated by Waters et al., 1995) corresponds to the different barrel rows indicated by arrows. **(C)** Receptive fields of neurons recorded in the sites numbered in **(A,B)**. The complete map of this experiment is represented in **Figure 7A**.

**FIGURE 7:**
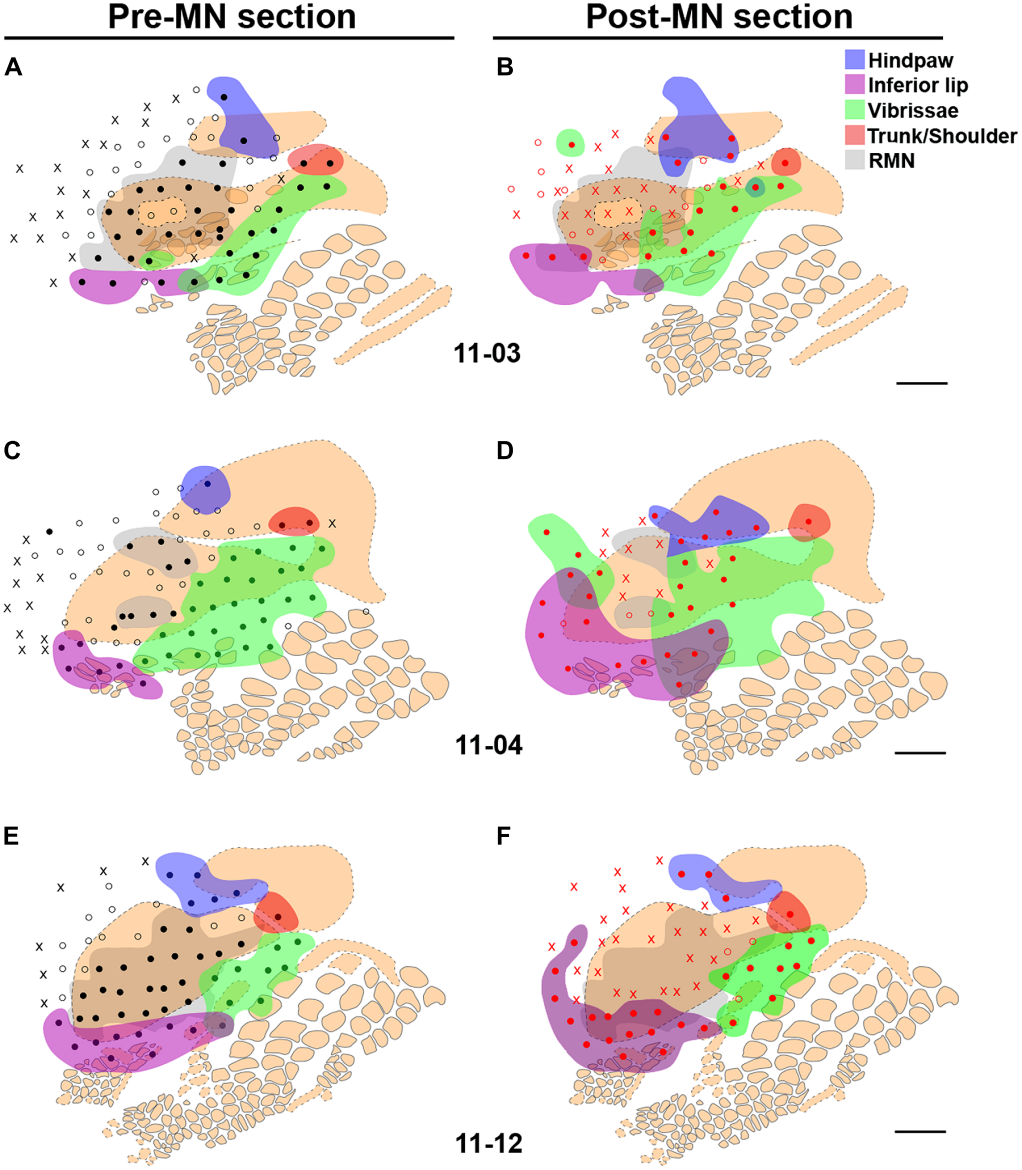
**Cutaneous representation in sites surrounding the RMN, and the immediate modification of neuronal responses following MN transection, in three cases of the *MN Intact* group: 11-03 **(A,B)**, 11-04 **(C,D)**, and 11-12 **(E,F)**. (A,C,E)** Same conventions as in **Figure 4** are adopted here. Note that the RMN (transparent gray zone) and the FBS (in beige) correspond grossly to the same cortical region. Colored splotches are regions containing neurons activated by stimulation of the inferior lip (purple), vibrissae (green), hind paw (blue), or the shoulder/neck region (red). **(B,D,F)** After mapping the RMN, the MN was then transected and the same cortical region was then remapped (red circles and “X”s). In the absence of inputs from the forepaw, most of the neurons in the RMN became unresponsive (red “X”s); but in a few sites neurons that were previously responsive to forepaw stimulation became activated by light cutaneous stimuli delivered to the face, shoulder, trunk, and/or hindpaw, depending on the location of the recording site (colored splotches “invading” the gray zone). Scale bar = 1 mm.

After the mapping sessions, tangential sections of S1 were processed for CO, revealing barrel fields (Wong-Riley and Welt, 1980) that marked the anatomical localization of the representation of different body parts (Chapin and Lin, 1984). In most of our experiments, we were able to identify the FBS as a region of diffuse CO reactivity in the expected location for this barrel field (**Figures 4**–**7**). We observed that inputs from the forepaw, carried by the isolated MN, activated most of the FBS (**Figures 4**,**5A**,**6**, and **7A,C**). Occasionally, neurons in some sites in the FBS were not considered part of the RMN because they only responded to deep stimulation of the forepaw (open circles in **Figures 5A**,**6**, and **7A,C,E**), or to superficial cutaneous stimulation of other body parts (e.g., cases 11-09 and 11-10 of the *PCL+MSC* group illustrated in **Figures 4D,F**, respectively).

Individual barrels inside the FBS were identified in cases 11-03 and 11-13. In case 11-03 from the *MN Intact* group (**Figure 6**), barrels inside the FBS were organized in four rows, corresponding to the representation of D2–D5 respectively, as demonstrated by Waters et al. (1995). However, in our recording conditions in which all brachial plexus nerves were sectioned with the exception of the MN, the more medial and posterior barrel row that would represent D5 contained neurons responsive to D4 or to the dorsal forepaw (sites #5 and #4, respectively in **Figure 6**). The two other barrel rows corresponding respectively to D3 and D4 had neurons responsive to P3 only (sites #2 and #3, respectively in **Figure 6**).

In some cases of the *MN Intact* and *PCL+MSC* groups, we found neurons responsive to superficial stimulation of the forepaw (and thus being part of the RMN) located outside the boundaries of the FBS (**Figures 4A,D,F,H** and **7A**). Part of those neurons was located in a region anterior to the FBS corresponding to the motor cortex (**Figures 4A,D–F**).

The cortex surrounding the RMN was usually responsive to somatosensory stimulation. The region anterior to the RMN was usually activated by deep stimulation of the forepaw (**Figures 5B,C** site #1) or by vibrissae stimulation. Medial and lateral to the RMN, we found neuronal responses to cutaneous stimulation of the hind paw and inferior lip, respectively (**Figures 5B,C** and **6**). Finally, we were able to map neuronal responses to vibrissae stimulation in the dysgranular cortical region previously identified as the “non-responsive zone” of rat S1 (Chapin and Lin, 1984), located immediately caudal to the RMN (blue zone in **Figure 5B** and green splotches in **Figure 7**).

### IMMEDIATE MODIFICATION OF NEURONAL RESPONSES IN THE RMN AS A RESULT OF TOTAL FORELIMB DEAFFERENTATION

After mapping the RMN in the *MN Intact* group, the MN itself was transected and the same cortical region was remapped (**Figure 7**). **Table 1** lists the number of sites containing neurons that changed their receptive fields after the lesion in three of the experiments.

As expected, without the inputs from the forepaw, neurons in many sites in the RMN became unresponsive to somatosensory stimulation (red “X”s in **Figures 7B,D,F**). However, in some recording sites in the previously defined RMN, neurons that were responsive to light cutaneous stimulation of the forepaw became responsive to the inferior lip, vibrissae, or the hind paw, depending on the location of the site. For instance, before MN transection in case 11-12 (**Figures 5** and 7E) the most lateral row of sites inside the RMN presented neurons that were responsive to D1 and Th. After MN transection, neurons in these sites became responsive to the inferior lip (**Figure 7F**). Curiously, the representation of the inferior lip in this case seemed to have expanded not only toward the RMN, but also to the region anterior to the RMN, where neurons were previously unresponsive or responsive to deep stimulation of the forepaw (**Figures 7E,F**). A similar expansion of the representation of the inferior lip after MN transection was also observed in case 11-04 (**Figures 7C,D**).

## DISCUSSION

In this study, we showed that the tubulization treatment with a PCL conduit, in which MSC were engrafted, resulted in a repaired MN with a higher number of MFs than when the repair was promoted by the PCL tube alone. Additionally, although the cortical region occupied by the RMN was larger in normal rats than in rats that had their MN sectioned and repaired 10 weeks before, the combination the two pro-regenerative strategies induced a larger cortical RMN than when only the PCL tube was used to treat the lesion. We additionally demonstrated that most of the FBS could be activated by MN inputs; and that, when isolated from the other brachial plexus inputs, the cortical representation of the MN did not present a clear topographic organization. Acute deprivation of these inputs led to immediate plastic modifications, in which the FBS became partially invaded by inputs from adjacent body parts. The significance and possible mechanisms underlying such results are discussed below.

### INFLUENCE OF MN INPUTS IN THE ORGANIZATION OF RAT S1

We showed here that the rat MN carried inputs from most of the anterior forepaw, with the exception of the D5. In primates, the MN innervates the radial side of the glabrous skin of the hand plus a small portion of the dorsal hand mainly restricted to distal phalanxes of D2 and D3 (Merzenich et al., 1983a,b; Planitzer et al., 2014). The territory innervated by the rat MN was thus similar to the one found in primates except for the wider innervation of the dorsal forepaw (Merzenich et al., 1983b).

The innervation provided by the MN presented some variability when different individuals were compared, especially when considering the dorsal forepaw and the HTh. Although the MN innervated a wide area of the anterior forepaw, different portions of the forepaw were not equally represented in the RMN, suggesting that sensory fibers originating in glabrous D1 and D2, Th and P1 were more numerous in the rat MN than fibers innervating other forepaw regions. Additionally, fibers originating in glabrous D1 and D2, Th and P1 seemed to predominate in the repaired MN, 10 weeks after the treatment.

In the normal adult rat, barrels in the FBS can be regarded as reliable anatomical markers for locating physiological representations of cutaneous inputs from the forepaw (Waters et al., 1995). One would thus presume that barrel rows in the posterior half of the FBS representing inputs coming from forepaw regions different from D1, D2, Th, and P1 would be silenced in our preparation. However, we showed that, when all brachial plexus nerves, with the exception of the MN, are acutely severed, most of the FBS could still be activated by inputs carried by the MN alone. Actually, it has been demonstrated that receptive fields of individual neurons in the rat forepaw representation of S1 can be large, encompassing multiple fingers and the palm, thus revealing a functional connectivity between the representation of different parts of the forepaw that are relatively far apart (Tutunculer et al., 2006). Additionally, in the somatosensory system, it is well established that cortical and subcortical circuits, especially at the level of cuneate nucleus (Xu and Wall, 1999) and thalamus (Nicolelis et al., 1993), receive subthreshold inputs that become suprathreshold right after interrupting the flow of cutaneous information from the periphery (for a review, see Calford, 2002; and Jones, 2000). Thus, after transection of the other brachial plexus nerves, subthreshold inputs relayed by the MN might become suprathreshold, thus promoting neuronal responses in the cortical regions previously activated by inputs carried by the sectioned nerves. This would expand the “normal threshold” representation of the MN and, at the same time, disrupt the topographic organization normally observed when all peripheral nerves are intact (Waters et al., 1995). For example, in our preparation, D4 inputs carried by the MN activated neurons in the D5 barrel row suggesting that the original topographic organization found in the FBS was modified by the lesion of the other brachial plexus nerves (especially the ulnar nerve). This additionally suggests that MN inputs relayed to some parts of the FBS (like inputs from D4 to the D5 barrel row) are probably sub-threshold in normal mapping conditions when cortical neurons are recorded with all nerves intact. Actually, convergence of inputs carried by different brachial plexus nerves to a same region of the rat somatosensory cortex was demonstrated by an fMRI study performed by Cho et al. (2008). Most likely, the dynamic balance of all inputs (either threshold or sub-threshold) provided by the different brachial plexus nerves to a same cortical region shapes the responses of individual neurons, thus promoting the emergence of the topographically organized map usually observed in the somatosensory cortex.

Similarly, neurons in the anterior half of the FBS processing MN inputs from D1, Th, P1, and D2 should receive subthreshold inputs carried by other peripheral nerves from adjacent body parts. Thus, after irreversible damage of the MN, neuronal responses to stimulation of the face, snout, and those parts of the forepaw innervated by the ulnar and radial nerves might become permanent in the anterior FBS, a well-known response to deafferentation that we observed after the acute MN lesion that we performed at the end of the mapping sessions in the *MN Intact* group (Merzenich et al., 1983b; Jones, 2000; Pearson et al., 2001, 2003; Cho et al., 2008; Weng et al., 2011). Since, in the other two experimental groups, damage to the MN had been reversed by the treatment with PCL, most of the responses to trigeminal and/or ulnar nerve inputs in the cortical territory normally activated by MN inputs disappeared after the regeneration of MN fibers. Information relayed by these regenerated fibers was then capable to reconquer part of its original cortical territory, corresponding to the RMN observed in our experimental groups. The more effective regeneration observed in the *PCL+MSC* group (see below) thus grants a larger reversion of the expansion of the representation of the adjacent body parts, reflected in the larger RMN measured 10 weeks after the treatment.

### IMPACT OF THE NERVE REPAIR STRATEGIES USED IN THIS STUDY

Besides characterizing the representation of inputs from the normal MN in S1, we also investigated the cortical representation of a repaired MN that was treated by the use of PCL conduits engrafted or not with MSC. Our working hypothesis was that the combined use of these two pro-regenerative strategies would optimize nerve regrowth, thus improving the reestablishment of physiological responses relayed to S1.

Our results suggest that modifications in body representation induced by peripheral damage can be reversed by the nerve tubulization treatment. Indeed, Wall et al. (1986) demonstrated that, after a MN transection, direct suture of the stumps promotes reinnervation of the hand and S1 reactivation. However, the original organization of the representation of the hand is replaced by a new one characterized by major topographical changes; additionally, sensorimotor function can be permanently impaired (Wall et al., 1986).

We show here that even a severe traumatic MN lesion involving tissue loss can be repaired up to the point in which a partial reestablishment of the damaged nerve representation can be achieved in S1. The repair strategy with a PCL tube permitted skin reinnervation capable of re-activating neurons in the CNS. The injection of MSC seems to increment this cortical reactivation, as the *PCL+MSC* group tended to present larger extents of RMN as compared with the *PCL-Only* group. This finding can be interpreted as a direct effect of a more efficient peripheral recovery resulting in a higher number of total MFs in the *PCL+MSC* group as compared with the *PCL-Only* group. Indeed, this result confirms our previous study showing that MN repair by means of PCL conduit combined with MSC-based therapy improves nerve regeneration, leading to functional recovery following a traumatic lesion (Oliveira et al., 2010). This better recovery was attributed to an increase in the number of MFs induced by MSC. Indeed, MSC can act as “mini-pumps” in the injured nerve microenvironment by secreting many growth-promoting molecules. For instance, MSC cocultured with dorsal root ganglion explants showed enhanced neurite outgrowth and neuronal cell survival via a production of an array of soluble factors. These cells were able to secrete a variety of proteins, including four proteins belonging to the family of neurotrophic factors: basic fibroblast growth factor (bFGF), nerve growth factor (NGF), ciliary neurotrophic factor (CNTF), and brain-derived neurotrophic factor (BDNF; Gu et al., 2010). Our group also observed high levels of NGF in the regenerated nerve treated with collagen conduit repair plus MSC, which presented higher number of MFs compared with the group treated with conduit repair only (Pereira Lopes et al., 2006). In addition to this paracrine effect, occurring either directly on nerve fibers or indirectly by modulating the behavior and/or phenotype of Schwann cells, MSC may support glial cell survival, axonal regrowth to the target organ and axonal re-myelination. Indeed, the therapeutic approach with MSC was able to elevate the protein levels of neurofilament and myelin basic protein as well as the expression of protein zero and peripheral myelin protein 22, which are thought to be specific and essential for proper myelination in Schwann cells (Chen et al., 2007).

The *PCL+MSC* group not only presented more MFs than *PCL-Only* group, it also presented a higher percentage of MFs in the optimum g-ratio (Ronchi et al., 2009). When the *PCL+MSC* and the *PCL-Only* groups were compared, we did not find differences in the percentage of MFs in the diameter range corresponding to Aβ fibers, which are responsible for mechanoception. However, in the fiber diameter range of 3.00–3.99 μm, which might correspond to A fibers (Sanders and Zimmermann, 1986), we observed a higher percentage of MFs in the *PCL-Only* as compared to the *PCL+MSC* group. It is possible that, independently of the fiber diameter distribution in the regenerated MN, the absolute number of MFs in each fiber diameter range was always higher in the *PCL+MSC* group than in the *PCL-Only* group. The other possibility is that besides contributing to a greater number of MFs in the regenerated MN, MSC might also contribute to increase the number of unmyelinated fibers, which are polymodal fibers, thus also displaying a role in the mechanoception (Craig, 2003). Indeed, in a previous work we found that MSC, not only increased the number of MFs in the regenerated mouse MN, but also the number of unmyelinated fibers (Oliveira et al., 2010).

Nerves repaired with the aid of MSC therapy might be relaying information from a larger skin region due to better nerve regrowth (represented by a larger number of MFs), thus promoting a larger area of cortical activation (a larger RMN) in S1, when compared with PCL treatment without MSC. Nonetheless, as demonstrated by Wall et al. (1986), the reactivated cortical area may not necessarily represent the original skin patch that was denervated by the lesion. Skin reinnervation may be incomplete and restricted to a small portion of the previously denervated region. The small reinnervated skin patch can be repeatedly represented in S1, occupying a large (and abnormal) area of cortical representation (Kaas et al., 1983). Indeed, the evidence provided in the present study supports the interpretation that the reinnervation that occurred 10 weeks after the peripheral nerve lesion was incomplete, since the RMN in animals from the *PCL-Only* and *PCL+MSC* groups were smaller and presented more restricted composite receptive fields than those from the *MN Intact* group. Additional mapping studies associated with multiple tracer injections into the reinnervated skin are needed to clarify this issue. Nonetheless, the results of the current study indicate that after combining pro-regenerative strategies to repair a severe peripheral nerve lesion, the cortical representation of the skin innervated by this nerve was larger, possibly due to an increase in the inputs from the skin sensory receptors as a result of a better regeneration of MFs. These results are of great relevance in the regenerative medicine field and, we hope, will encourage the implementation of these new therapeutic strategies in the human clinical set.

## Conflict of Interest Statement

The authors declare that the research was conducted in the absence of any commercial or financial relationships that could be construed as a potential conflict of interest.
